# Increased Risk of Clinically Significant Gallstones following an Appendectomy: A Five-Year Follow-Up Study

**DOI:** 10.1371/journal.pone.0165829

**Published:** 2016-10-27

**Authors:** Shiu-Dong Chung, Chung-Chien Huang, Herng-Ching Lin, Ming-Chieh Tsai, Chao-Hung Chen

**Affiliations:** 1 Department of Surgery, Far Eastern Memorial Hospital, Banciao, Taipei, Taiwan; 2 Graduate Program in Biomedical Informatics, College of Informatics, Yuan-Ze University, Chung-Li, Taiwan; 3 Sleep Research Center, Taipei Medical University Hospital, Taipei, Taiwan; 4 School of Health Care Administration, Taipei Medical University, Taipei, Taiwan; 5 Division of Gastroenterology, Department of Internal Medicine, Cathay General Hospital, Hsinchu Branch, Taiwan; 6 Department of Cosmetic Applications and Management, Mackay Junior College of Medicine, Nursing, and Management, Taipei, Taiwan; 7 Department of Thoracic Surgery, MacKay Memorial Hospital, Taipei, Taiwan; 8 Department & Institute of Physiology, National Yang-Ming University, Taipei, Taiwan; Osaka University Graduate School of Medicine, JAPAN

## Abstract

Although the vermiform appendix is commonly considered a vestigial organ, adverse health consequences after an appendectomy have garnered increasing attention. In this study, we investigated the risks of gallstone occurrence during a 5-year follow-up period after an appendectomy, using a population-based dataset. We used data from the Taiwan Longitudinal Health Insurance Database 2005. The exposed cohort included 4916 patients who underwent an appendectomy. The unexposed cohort was retrieved by randomly selecting 4916 patients matched with the exposed cohort in terms of sex, age, and year. We individually tracked each patient for a 5-year period to identify those who received a diagnosis of gallstones during the follow-up period. Cox proportional hazard regressions were performed for the analysis. During the 5-year follow-up period, the incidence rate per 1000 person-years was 4.71 for patients who had undergone an appendectomy, compared to a rate of 2.59 for patients in the unexposed cohort (*p*<0.001). Patients who had undergone an appendectomy were independently associated with a 1.79 (95% CI = 1.29~2.48)-fold increased risk of being diagnosed with gallstones during the 5-year follow-up period. We found that among female patients, the adjusted hazard ratio of gallstones was 2.25 (95% CI = 1.41~3.59) for patients who underwent an appendectomy compared to unexposed patients. However, for male patients, we failed to observe an increased hazard for gallstones among patients who underwent an appendectomy compared to unexposed patients. We found an increased risk of a subsequent gallstone diagnosis within 5 years after an appendectomy.

## Introduction

Acute appendicitis, or inflammation of the appendix, is among the most frequently occurring abdominal emergencies in the industrialized world. The overall incidence of appendicitis is 75~120 per 100,000 persons per year in Western populations and is 108 per 100,000 persons per year in Taiwan [1–4], with a higher rate among males than females. Although it is controversial, suspicion of appendicitis is preferably managed with a clinical assessment and a liberal attitude to early surgical exploration, i.e., an appendectomy, to avoid life-threatening complications such as peritonitis and a ruptured appendix [5,6].

Although the vermiform appendix in humans is commonly considered a vestigial organ, a certain immune function is believed to be involved based on its association with substantial lymphatic tissues. Findings suggest that the appendix is well-suited to serve as a “safe house” for biofilm formation to preserve and protect commensal bacteria needed in the colon [7]. More evidence has accumulated associating an appendectomy with chronic inflammatory disorders of the gastrointestinal (GI) tract closer to the appendix, such as ulcerative colitis [8,9] and Crohn’s disease [10,11]. Cancer risks following an appendectomy were also observed in GI tract systems including colon cancer and the reproductive system including prostate cancer [12,13].

Gallstone disease is one of the most frequent and costly of all digestive diseases [14]. In the adult western population, about 10%~15% of people present with gallstones. Between 1% and 4% of these individuals develop symptoms within a year. In addition to an appendectomy, acute cholecystitis is another common acute surgical condition of the abdomen [15,16]. Appendicitis and removal of the appendix are proposed as being linked to the pathogenesis of gallstones. For example, due to inflammation, ulceration of the mucosa of the appendix occurs during appendicitis, which enables bacterial translocation from the appendix to nearby structures [17,18]. The roles of bacterial infections and inflammatory processes have also received attention in the formation of gallstones [19]. Although an appendectomy was observed to be associated with certain GI track illnesses, no one has specifically examined its relationship with gallstones.

Thus, the aim of this study was to use a nationwide population-based dataset to investigate whether an appendectomy increases the risk for a subsequent gallstone diagnosis during a 5-year follow-up period. Both males and females were explicitly examined.

## Methods

### Database

We obtained the data of the study sample from the Longitudinal Health Insurance Database 2000 (LHID2005). Taiwan initiated its National Health Insurance (NHI) program in 1995, and the coverage rate has been about 98.4% since its beginning. The LHID2005 consists of registration files and original medical claims for 1,000,000 enrollees randomly selected from all enrollees listed in the 2005 Registry of Beneficiaries under the NHI program (*n* = 23.72 million) by the Taiwan National Health Research Institutes. The LHID2005 enables researchers in Taiwan to longitudinally follow-up the utilization of medical services for these selected 1,000,000 enrollees. This study was exempt from full review by the Institutional Review Board (TMU-JIRB 201412035) since the LHID2005 consists of de-identified secondary data released to the public for research purposes.

### Study Sample

This retrospective cohort study included an exposed cohort and an unexposed cohort. To select the exposed cohort, we first identified 6252 patients who were hospitalized and underwent an appendectomy (ICD-9-CM procedure code 470, 470.1, or 470.9) between January 1, 2002 and December 31, 2008 from the LHID2005. We excluded 1219 patients aged <18 years in order to limit the study sample to the adult population. We assigned the date of the appendectomy as the index date for the study cohort. We further excluded patients who had a history of gallstones (ICD-9-CM) code 574.0~574.4 or 574.6~574.9) before their index date (*n* = 117). As a result, the exposed cohort included 4916 subjects who had undergone an appendectomy.

As for the unexposed cohort, we first excluded all patients who had undergone an appendectomy since 1995. The Taiwan NHI began in 1995 and the LHID2005 did not allow us to trace medical claims before 1995. Therefore, we cannot exclude the possibility that some selected comparison patients might have undergone an appendectomy before 1995. However, this potential bias would have led the results toward the null. Thereafter, we randomly retrieved 4916 patients (one for every patient who underwent an appendectomy) to match the exposed cohort in terms of sex, age group (<30, 30~39, 40~49, 50~59, 60~69, and >69 years), and the year of the index date using the SAS proc surveyselect program (SAS System for Windows, vers. 8.2, SAS Institute, Cary, NC). The year of the index date was the year in which they underwent an appendectomy for the exposed cohort. However, the unexposed patients were selected by matching them to a given patient who underwent an appendectomy simply on their utilization of medical services in the same index year of that particular exposed patient. Furthermore, we defined their first healthcare use occurring in the index year as their index date for the unexposed cohort. Correspondingly, we likewise ensured that none of the selected unexposed patients had a history of gallstones before their index date. We further assured that none of the unexposed patients underwent an appendectomy during the 5-year follow-up period. As a result, 9832 sampled patients were included in this study.

We individually tracked each subject for a 5-year period from their index date to identify those who had received a diagnosis of gallstones during the follow-up period. In order to assure the gallstone diagnostic validity, we only counted those patients who had received two or more gallstones diagnoses.

### Statistical analysis

We used the SAS system for statistical analyses. Pearson Chi-squared tests were carried out to compare differences between patients who underwent an appendectomy and comparison subjects in terms of monthly income (NT$0~15,840, NT$15,841~25,000, ≥NT$25,001; the average exchange rate in 2007 was US$1.00≈New Taiwan (NT)$29), geographical location, and urbanization level of the subject’s residence (five levels with 1 being the most urbanized and 5 being the least). We further used the Kaplan-Meier method and a log-rank test to examine the difference in 5-year gallstone-free survival rates between patients who did and did not undergo an appendectomy. In addition, we further performed stratified Cox proportional hazard regressions (stratified by sex, age group, and the year of the index date) to calculate the hazard ratio (HR) and its corresponding 95% confidence interval (CI) for the subsequent occurrence of gallstones during the 5-year follow-up period between patients who did and did not undergo an appendectomy. We also took medical co-morbidities including hyperlipidemia (ICD-9-CM codes 272.0–272.4), diabetes (ICD-9-CM codes 250), hypertension (ICD-9-CM codes 401–405), coronary heart disease (ICD-9-CM codes 410–414 or 429.2) and obesity (ICD-9-CM codes 278, 278.0, 278,00 or 278.01) into consideration in the regression models. In this study, we found that the proportional hazards assumption was satisfied since the survival curves for both strata (patients who did and did not undergo an appendectomy) had hazard functions that were proportional over time. Furthermore, we censored those subjects who died or who were lost to follow-up during that time (457 from the study cohort (9.3% of the subjects who underwent an appendectomy) and 437 from the comparison cohort (9.0% of the comparison subjects)). We used a significance level of 0.05.

## Results

Distributions of demographic and clinical characteristics stratified by the presence or absence of an appendectomy are presented in Table 1. After being matched for sex, age group, and the year of the index date, we found that there was a significant difference in monthly income (*p*<0.001) and geographic region (*p* = 0.005) between patients who underwent an appendectomy and unexposed patients. Furthermore, we found that patients who underwent an appendectomy had a higher prevalence of hypertension (*p* = 0.002), coronary heart disease (*p*<0.001), hyperlipidemia (*p*<0.001), and diabetes (*p*<0.001) than unexposed patients. However, we failed to observe a significant difference in urbanization level between patients who underwent an appendectomy and unexposed patients (*p* = 0.971).

**Table 1:** Demographic and clinical characteristics of sampled subjects (N = 9832).

Variable	Subjects who underwent an appendectomy *N* = 4916		Comparison subjects *N* = 4916		*p* value
	Total no.	Column %	Total no.	Column %	
Male	2612	53.1	2612	53.1	>0.999
Age group (years)					>0.999
18~29	1622	33.0	1622	33.0	
30~39	1169	23.8	1169	23.8	
40~49	899	18.3	899	18.3	
50~59	553	11.2	553	11.2	
60~69	359	7.3	359	7.3	
>69	314	6.4	314	6.4	
Urbanization level					0.971
1 (most)	1510	30.7	1492	30.3	
2	1403	28.5	1422	28.9	
3	833	16.9	832	16.9	
4	658	13.4	645	13.1	
5 (least)	512	10.4	525	10.7	
Monthly income					<0.001
NT$0~15,840	1828	37.2	2125	43.2	
NT$15,841~25,000	1840	37.4	1519	30.9	
≥NT$25,001	1248	25.4	1272	25.9	
Geographic region					0.005
Northern	2328	47.4	2361	48.0	
Central	1115	22.7	1196	24.3	
Southern	1337	27.2	1266	25.8	
Eastern	136	2.8	93	1.9	
Hypertension	989	20.1	866	17.6	0.002
Diabetes	489	10.0	388	7.9	<0.001
Coronary heart disease	486	9.9	327	6.7	<0.001
Hyperlipidemia	874	17.8	732	14.9	<0.001
Obesity	51	1.0	39	0.8	0.204

Note: The average exchange rate in 2008 was US$1.00≈New Taiwan (NT)$29.

**Fig 1:**
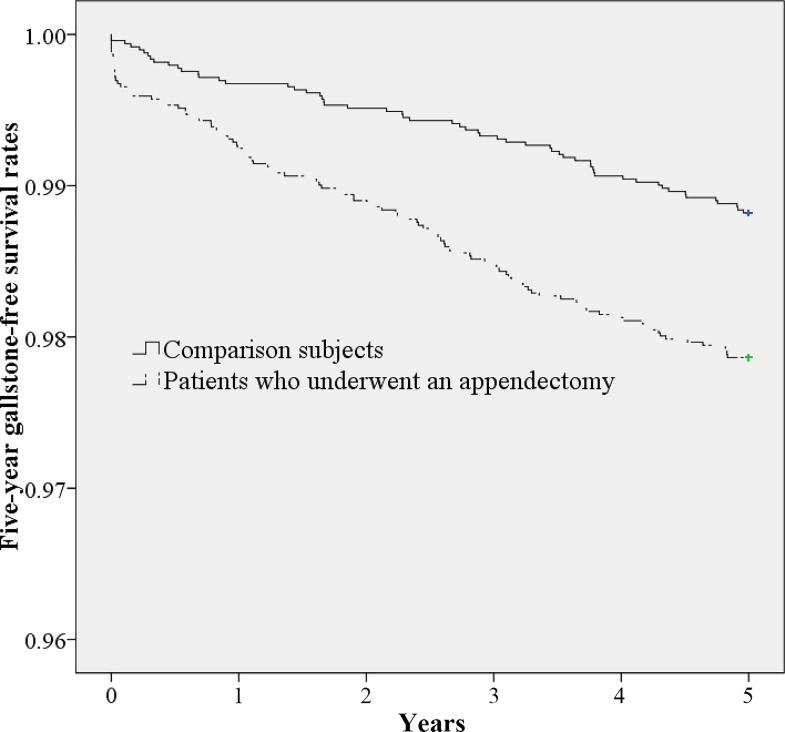
Five-year gallstone-free survival rates for those who underwent an appendectomy and comparison subjects.

Table 2 shows the incidence of gallstones during the 5-year follow-up period. Incidence rates of gallstones during the 5-year follow-up period were 4.71 (95% CI: 3.85~5.70) and 2.59 (95% CI: 1.97~3.35) per 1000 person-years for the exposed and unexposed cohorts, respectively. The log-rank test revealed that patients who underwent an appendectomy were more likely to have gallstones than unexposed patients (*p*<0.001). Gallstone-free survival curves between patients who underwent an appendectomy and unexposed patients are presented in Fig 1.

**Table 2:** Crude and covariate-adjusted hazard ratios (HRs) for gallstones among sampled subjects during the 5-year follow-up period.

Presence of gallstones	Total sample *(N* = 9832)	Subjects who underwent an appendectomy (*N* = 4916)	Comparison subjects *(N* = 4916)
Five-year follow-up period			
Yes, *n* (%)	163 (1.66)	105 (2.14)	58 (1.18)
Incidence rate per 1000 person-years (95% CI)	3.65 (3.11~4.25)	4.71 (3.85~5.70)	2.59 (1.97~3.35)
Crude HR (95% CI)	-	1.83*** (1.32~2.53)	1.00
Adjusted ^a^ HR (95% CI)	-	1.79*** (1.29~2.48)	1.00

Notes: CI, confidence interval. The HR was calculated by a stratified Cox proportional hazard regression which was stratified by sex, age group, and the year of the index date.

^a^ Adjustments were made for subjects’ monthly income, geographic region, hypertension, coronary heart disease, hyperlipidemia, and diabetes

*** p<0.001.

Table 2 also shows the crude and adjusted HRs for gallstones by cohort. The stratified Cox proportional analysis (stratified by age, sex, and the year of the index date) suggested that the HR for gallstones during the 5-year follow-up period was 1.79 (95% CI = 1.29~2.48) for patients who underwent an appendectomy compared to unexposed patients after censoring individuals who died during the follow-up period and adjusting for patients’ monthly income, geographic region, hypertension, coronary heart disease, hyperlipidemia, and diabetes.

Table 3 further analyzes the HRs of gallstones by cohort according to sex. We found that among female patients, the adjusted HR of gallstones was 2.25 (95% CI = 1.41~3.59) for patients who underwent an appendectomy compared to unexposed patients. However, for male patients, we failed to observe an increased hazard for gallstones among patients who underwent an appendectomy compared to unexposed patients.

**Table 3:** Crude and covariate-adjusted hazard ratios (HRs) for gallstones among sampled subjects during the 5-year follow-up period by sex.

Presence of gallstones	Sex			
	Males		Females	
	Subjects who underwent an appendectomy *N* = 2612	Comparison subjects *N* = 2612	Subjects who underwent an appendectomy *N* = 2304	Comparison subjects *N* = 2304
Five-year follow-up period				
Yes, *n* (%)	43 (1.65)	32 (1.23)	62 (2.69)	26 (1.12)
Incidence rate per 1000 person-years (95% CI)	3.45 (2.50–4.65)	2.57 (1.76–3.63)	5.64 (4.32–7.22)	2.36 (1.54–3.46)
Crude HR (95% CI)	1.34 (0.85~2.13)	1.00	2.43***(1.53~3.86)	1.00
Adjusted HR^a^ (95% CI)	1.38 (0.87~2.20)	1.00	2.25***(1.41~3.59)	1.00

Notes: CI, confidence interval. The HR was calculated by a stratified Cox proportional hazard regression which was stratified by age group and the year of the index date.

^a^ Adjustments were made for subjects’ monthly income, geographic region, hypertension, coronary heart disease, hyperlipidemia and diabetes

*** p<0.001.

We further performed a sensitivity analyses to calculate the HRs of gallstones by cohort according to the diagnosis number of gallstones during the 5-year follow-up period (Table 4). We found that the relationship between appendectomy and risk for a subsequent gallstone still sustained regardless of the diagnosis number of gallstones during the 5-year follow-up period.

**Table 4:** Sensitivity analysis.

Presence of gallstones	Total sample *(N* = 9832)	Subjects who underwent an appendectomy (*N* = 4916)	Comparison subjects *(N* = 4916)
Patients who received ≥ 1 gallstones diagnosis during the follow-up period	238 (2.4)	166 (3.4)	72 (1.5)
Yes			
Crude HR (95% CI)	—	2.35*** (1.78–3.11)	1.00
Adjusted ^a^ HR (95% CI)	—	2.25*** (1.70–2.99)	1.00
Patients who received ≥3 gallstones diagnoses during the follow-up period			
Yes	92 (0.94)	59 (1.20)	33 (0.67)
Crude HR (95% CI)	-	1.80*** (1.17–2.76)	1.00
Adjusted ^a^ HR (95% CI)	-	1.71** (1.11–2.64)	1.00
Patients who received ≥4 gallstones diagnoses during the follow-up period			
Yes	63 (0.63)	40 (0.81)	23 (0.47)
Crude HR (95% CI)		1.66** (1.15–2.39)	1.00
Adjusted ^a^ HR (95% CI)		1.60* (1.10–2.31)	1.00

Notes: CI, confidence interval. The HR was calculated by a stratified Cox proportional hazard regression which was stratified by sex, age group, and the year of the index date.

^a^ Adjustments were made for subjects’ monthly income, geographic region, hypertension, coronary heart disease, hyperlipidemia, and diabetes

*** p<0.001

** p<0.01

* p<0.05.

## Discussion

This is the first report of the risk of gallstones following an appendectomy. Our population-based study showed that during the 5-year follow-up period, incidence rates per 1000 person-years were 4.71 and 2.59 for patients who underwent an appendectomy and patients in the unexposed cohort, respectively. After adjusting for age, sex, year, monthly income, geographic region, hypertension, coronary heart disease, hyperlipidemia, and diabetes, patients who underwent an appendectomy were independently associated with a 1.79-fold increased risk of having been diagnosed with gallstones during the 5-year follow-up period. In terms of sex stratification, risks of gallstones within 5 years specifically increased for females, demonstrating a significant hazard ratio of 2.25.

The real function of the human appendix has long been argued. Although it might not be considered vital, recent studies have observed an association between removal of the appendix and GI tract diseases, such as an elevated risk for Crohn’s disease and a reduced risk for ulcerative colitis [8–11]. In a population-based study, increased risks of malignancy in digestive systems, such as colorectal cancer, were found following an appendectomy. Moreover, the incidence of gall bladder cancer increased 2-fold among patients who underwent an appendectomy. Nevertheless, there has been no report examining the association between appendicitis/an appendectomy and gallbladder stones.

The present study suggested that the risk of gallstones was significantly higher for patients within 5 years after an appendectomy. It is worth attention that significantly higher proportions of patients with appendectomy reported of hypertension, coronary heart disease, hyperlipidemia and diabetes in bivariate analyses. The exposed cohort might be presumed to have greater risks of developing cholelithiasis compared to the unexposed cohort. However, the differences between these two groups were minimal (only 2–3%) and the results of increasing risks of following gallstones among patients with appendectomy remained significant after the diseases of hypertension, coronary heart disease, hyperlipidemia and diabetes were considered in the multivariate regression models for adjustment.

In addition, the results were even more profound among females (with an adjusted HR of 2.25). Due to exposure to estrogen and progesterone, women indeed present a higher prevalence of gallstones than men [20]. Obesity is an established risk factor for the formation of cholesterol gallstones, and the risk is especially high in women [21]. The specific pathway linking an appendectomy to gallstone formation, especially in women, deserves attention and further study. However, due to smaller sample sizes in our analyses stratified by sex, statistical power might be restricted. Future more extensive investigations possibly collecting laboratory investigations in longitudinal follow-up study design with larger sample sizes are warranted to further ascertain the link between appendectomy and its consequent risks on gallstones.

How appendicitis or an appendectomy is associated with the formation of gallstones is not known, yet preliminary evidence suggests possibilities. It is proposed that bacterial infections may be involved which result in compromised excretion of bile acids and excessive bilirubin in the bile, facilitating the formation of gallstones. Specifically, due to inflammation, ulceration of the mucosa in the appendix occurs during appendicitis, which accelerates bacterial translocation from the appendix to the hepatic portal system [18]. In addition, the appendix is recognized as an essential component of mammalian mucosal immune function, particularly B-lymphocyte-mediated immune responses and extrathymically derived T-lymphocytes [22]. As one of the guardians of the internal body from the hostile external environment being removed after an appendectomy, the "safe house" for commensal bacteria is damaged. Impaired immunity may facilitate proliferation of pathogens which induce regional infectious diseases, such as Crohn’s disease [7,11]. Animal models also exhibit that when pathogens, such as the gram-negative *Escherichia coli* that frequently infects the appendix, move to hepatic tissues, the bacteria impede the hepatocyte microcirculation, producing damage to liver cells and compromising excretion of bile acids into the bile canaliculi [23]. Cholesterol supersaturation occurs when the cholesterol concentration in the bile exceeds the ability of the bile to hold it in solution, due to compromised excretion of bile acids. Then, crystals form and grow into stones.

In addition to reduced bile acids, excessive bilirubin also plays a role. Translocation of pathogens such as *E*. *coli* into serum may destroy red blood cells and provoke intravascular hemolysis, causing excessive bilirubin to circulate in the blood, which is associated with pigment stone formation [24]. Tabata and Nakayama also proposed that E. coli may impact the formation of bile pigment calcium stones by generating beta-glucuronidase and then deconjugating bilirubin diglucuronide to produce free unconjugated bilirubin. Gallstones develop when this is combined with calcium [25]. Indeed, in a population-based study from Denmark, elevated serum bilirubin levels were found to be associated with a risk of gallstone formation [26]. Similar pathways were suggested to link an appendectomy with increased risks of Crohn’s disease, which is subsequently considered to be a risk factor for gallstones [11,27,28].

Our findings have considerable implications. About 10%~15% of the adult population has gallstones. Gallstones and subsequent complications are the most common and costly of all digestive diseases and contribute to substantial financial burdens to healthcare systems [16,29]. Many patients with gallstones are asymptomatic or have mild, non-specific symptoms which may be attributed to other conditions. Misdiagnoses and unnecessary investigations and treatments may be applied. These impact patients’ quality of life and affect the allocation of healthcare resources [30]. Thus, more thoroughly considering risk factors for gallstones may help appropriately manage patients. We found that an appendectomy may be considered a potential risk factor for subsequent gallstones. Earlier attention to following gallstone risks may benefit patients with an appendectomy, as some risk and protective factors of gallstones are modifiable, such as dietary intake, physical activity, and obesity, and treatments to prevent symptoms and complications are available [30–35]. Appropriate attention and monitoring following an appendectomy could also help detect mild, non-specific symptoms of gallstones to promote appropriate diagnoses and management. Meanwhile, our findings further motivate a more-cautious consideration of the necessity of surgical interventions to remove the appendix, due to possible shorter- or longer-term health effects after the appendectomy.

Strengths of this study include the use of long-term population-based data with a low loss to follow-up rate. A longitudinal observation is then feasible, with selection and non-response biases being minimized. In using a claims dataset, we did take certain risk factors for gallstones, such as age, sex, hyperlipidemia [36,37], and diabetes [38], into consideration in estimating HRs for gallstones following an appendectomy.

However, several limitations of this study merit attention before our findings are considered. First, our claims database represents patients who had sought treatment for gallstones. Symptoms of gallstones may range from asymptomatic, mild, non-specific ones which are hard to diagnose, to severe pain and/or complications which are easily detected by health professionals. Although many patients may stay asymptomatic throughout their lifetime, around 1%~4% of them develop symptoms annually [15]. It is also possible that gallstones can be detected coincidentally due to other conditions [30]. Misdiagnosing symptomatic gallstones as appendicitis might be a concern. However, due to varied location of the pain, adequate physical examination to assess the site of the pain would differentiate the diagnoses of appendicitis and gallstones. Further with modern day imaging, the negative appendectomy rate is usually low [39]. In addition, our findings may potentially have been compromised by patients with an appendectomy possibly having greater healthcare utilization, thus increasing the likelihood of gallstone identification. However, postoperative visits after comparatively low-risk surgeries such as an appendectomy may be minimal [40]. Further, we performed sensitivity analyses, excluding patients receiving a diagnosis of gallstones within a month after an appendectomy (n = 19), to address this concern. The results were fairly consistent (with an adjusted HR of 1.56, *p* = 0.011). The relationship between appendectomy and the following increased risks of gallstones remained essential to consider.

Second, because the NHI program was initiated in 1995, patients who underwent an appendectomy prior to 1995 would have been classified as non-appendectomy patients in the analysis, which might possibly have biased our results towards the null. Third, as abdominal ultrasound data before appendicitis/appendectomy were not available, we were unable to determine whether cases had asymptomatic gallstones present before their appendectomies. It thus could not be ruled out that asymptomatic gallstones might increase the risk of appendicitis/appendectomy, or that shared characteristics of persons with asymptomatic gallstones and appendectomy might account for the association found. Fourth, in the main analysis, we only counted those patients who had received two or more gallstones diagnoses as gallstones cases in order to assure the diagnostic validity. In the calculation of incidence rates, those with a single diagnosis remained in the denominator but were not counted in the nominator. We thus had more conservative estimates of the incident rates. In the regression analysis, non-differential misclassification (i.e., when disease-status classification errors equal among exposed and unexposed groups) would bias the estimate of association towards the null. Further in sensitivity analyses in Table 4, similar findings were observed if we treated patients who received ≥ 1, ≥ 3 and ≥ 4 gallstones diagnosis during the follow-up period as having the outcomes of gallstone diseases. Finally, individual information such as physical activity, body-mass index, dietary habits, and family history and genetics, which may contribute to the formation of gallstones, were not available for risk adjustment in our claims dataset. In addition, we found appendectomy cases were more likely to have clinical diagnoses of hypertension, diabetes, coronary heart disease, and hyperlipidemia that might also be associated with gallstone disease. It is possible these factors were inadequately adjusted for with the available data consisting of clinical diagnoses found in medical records, rather than physical examination and laboratory screening.

In conclusion, we found that patients who underwent an appendectomy had significantly higher risks of a subsequent diagnosis of gallstones within 5 years, especially among women. Although replication is required to confirm these findings, we stress the importance of contemplating the imperativeness of a surgical intervention for appendicitis and the monitoring of short- and long-term health effects of patients after an appendectomy. Further clinical or basic studies are needed to elucidate whether gallstones are consequences or comorbidities of appendicitis/an appendectomy.

## References

[1] Blomqvist P, Ljung H, Nyrén O, Ekbom A. Appendectomy in Sweden 1989–1993 assessed by the Inpatient Registry. J Clin Epidemiol, 1998; 51: 859–865. 9762879 10.1016/s0895-4356(98)00065-1

[2] Buckius MT, McGrath B, Monk J, Grim R, Bell T, Ahuja V. Changing epidemiology of acute appendicitis in the United States: study period 1993–2008. J Surg Res. 2012; 175: 185–190. 10.1016/j.jss.2011.07.017 22099604

[3] Al-Omran M, Mamdani M, McLeod RS. Epidemiologic features of acute appendicitis in Ontario, Canada. Can J Surg. 2003; 46: 263–268. 12930102 PMC3211626

[4] Lin KB, Lai KR, Yang NP, Chan CL, Liu YH, Pan RH, et al Epidemiology and socioeconomic features of appendicitis in Taiwan: a 12-year population-based study. World J Emerg Surg. 2015; 10: 42 10.1186/s13017-015-0036-3 26388932 PMC4573493

[5] Velanovich V, Satava R. Balancing the normal appendectomy rate with the perforated appendicitis rate: implications for quality assurance. Am Surg. 1992; 58: 264–269. 1586087

[6] Andersson RE. Short and long-term mortality after appendectomy in Sweden 1987 to 2006. Influence of appendectomy diagnosis, sex, age, co-morbidity, surgical method, hospital volume, and time period. A national population-based cohort study. World J Surg. 2013; 37: 974–981. 10.1007/s00268-012-1856-x 23192168

[7] Randal Bollinger R, Barbas AS, Bush EL, Lin SS, Parker W. Biofilms in the large bowel suggest an apparent function of the human vermiform appendix. J Theor Biol. 2007; 249: 826–831. 10.1016/j.jtbi.2007.08.032 17936308

[8] Koutroubakis IE, Vlachonikolis IG. Appendectomy and the development of ulcerative colitis: results of a metaanalysis of published case-control studies. Am J Gastroenterol. 2000; 95: 171–176. 10.1111/j.1572-0241.2000.01680.x 10638578

[9] Koutroubakis IE, Vlachonikolis IG, Kouroumalis EA. Role of appendicitis and appendectomy in the pathogenesis of ulcerative colitis: a critical review. Inflamm Bowel Dis. 2002; 8: 277–286. 12131612 10.1097/00054725-200207000-00007

[10] Andersson RE, Olaison G, Tysk C, Ekbom A. Appendectomy is followed by increased risk of Crohn's disease. Gastroenterology. 2003; 124: 40–46. 10.1053/gast.2003.50021 12512028

[11] Kaplan GG, ackson T, Sands BE, Frisch M, Andersson RE, Korzenik J. The risk of developing Crohn's disease after an appendectomy: a meta-analysis. Am J Gastroenterol. 2008; 103: 2925–2931. 10.1111/j.1572-0241.2008.02118.x 18775018

[12] Ratanarapee S, Nualyong C. Acute appendicitis as primary symptom of prostatic adenocarcinoma: report of a case. J Med Assoc Thai. 2010; 93: 1327–1331. 21114214

[13] Lai HW, Loong CC, Tai LC, Wu CW, Lui WY. Incidence and odds ratio of appendicitis as first manifestation of colon cancer: a retrospective analysis of 1873 patients. J Gastroenterol Hepatol. 2006; 21: 1693–1696. 10.1111/j.1440-1746.2006.04426.x 16984591

[14] Everhart JE, Khare M, Hill M, Maurer KR. Prevalence and ethnic differences in gallbladder disease in the United States. Gastroenterology. 1999; 117: 632–639. 10464139 10.1016/s0016-5085(99)70456-7

[15] Gurusamy KS, Davidson C, Gluud C, Davidson BR. Early versus delayed laparoscopic cholecystectomy for people with acute cholecystitis. Cochrane Database Syst Rev. 2013; 6: CD005440 10.1002/14651858.CD005440.pub3 23813477

[16] Gallstones and laparoscopic cholecystectomy. NIH Consens Statement, 1992; 10: 1–28. 1301217

[17] Burcharth J, Pommergaard HC, Rosenberg J, Gögenur I. Hyperbilirubinemia as a predictor for appendiceal perforation: a systematic review. Scand J Surg. 2013; 102: 55–60. 10.1177/1457496913482248 23820677

[18] Sisson RG, Ahlvin RC, Harlow MC. Superficial mucosal ulceration and the pathogenesis of acute appendicitis. Am J Surg. 1971; 122: 378–380. 5570610 10.1016/0002-9610(71)90262-5

[19] Hoogerwerf WA, Soloway RD. Gallstones. Curr Opin Gastroenterol. 1999; 15: 442–447. 17023987 10.1097/00001574-199909000-00012

[20] Lee JY, Keane MG, Pereira S. Diagnosis and treatment of gallstone disease. Practitioner. 2015; 259: 15–19. 26455113

[21] Jorgensen T. Prevalence of gallstones in a Danish population. Am J Epidemiol. 1987; 126: 912–921. 3310613 10.1093/oxfordjournals.aje.a114728

[22] Zahid A. The vermiform appendix: not a useless organ. J Coll Physicians Surg Pak. 2004; 14: 256–258. 15228837

[23] Rink RD, Kaelin CR, Giammara B, Fry DE. Effects of live Escherichia coli and Bacteroides fragilis on metabolism and hepatic pO2. Circ Shock. 1981; 8: 601–611. 7026081

[24] Njeze GE. Gallstones. Niger J Surg. 2013; 19: 49–55. 10.4103/1117-6806.119236 24497751 PMC3899548

[25] Tabata M, Nakayama F. Bacteria and gallstones. Etiological significance. Dig Dis Sci. 1981; 26: 218–224. 7238247 10.1007/BF01391633

[26] Stender S, Frikke-Schmidt R, Nordestgaard BG, Tybjærg-Hansen A. Extreme bilirubin levels as a causal risk factor for symptomatic gallstone disease. JAMA Intern Med. 2013; 173: 1222–1228. 10.1001/jamainternmed.2013.6465 23753274

[27] Lapidus A, Bångstad M, Aström M, Muhrbeck O. The prevalence of gallstone disease in a defined cohort of patients with Crohn's disease. Am J Gastroenterol. 1999; 94: 1261–1266. 10.1111/j.1572-0241.1999.01076.x 10235204

[28] Parente F, Pastore L, Bargiggia S, Cucino C, Greco S, Molteni M, et al Incidence and risk factors for gallstones in patients with inflammatory bowel disease: a large case-control study. Hepatology. 2007; 45: 1267–1274. 10.1002/hep.21537 17464998

[29] Wang JK, Foster SM, Wolff BG. Incidental gallstones. Perm J. 2009; 13: 50–54. 10.7812/tpp/08-050 PMC3034431 21373230

[30] Internal Clinical Guidelines Team (UK). Gallstone Disease: Diagnosis and Management of Cholelithiasis, Cholecystitis and Choledocholithiasis. London: National Institute for Health and Care Excellence (UK); 2014. 25473723

[31] Attili AF, Scafato E, Marchioli R, Marfisi RM, Festi D. Diet and gallstones in Italy: the cross-sectional MICOL results. Hepatology. 1998; 27: 1492–1498. 10.1002/hep.510270605 9620318

[32] Tsai CJ, Leitzmann MF, Willett WC, Giovannucci EL. Fruit and vegetable consumption and risk of cholecystectomy in women. Am J Med. 2006; 119: 760–767. 10.1016/j.amjmed.2006.02.040 16945611

[33] Leitzmann MF, Giovannucci EL, Rimm EB, Stampfer MJ, Spiegelman D, Wing AL, et al The relation of physical activity to risk for symptomatic gallstone disease in men. Ann Intern Med. 1998; 128: 417–425. 9499324 10.7326/0003-4819-128-6-199803150-00001

[34] Mabee TM, Meyer P, DenBesten L, Mason EE. The mechanism of increased gallstone formation in obese human subjects. Surgery. 1976; 79: 460–468. 1257908

[35] Willett WC, Dietz WH, Colditz GA. Guidelines for healthy weight. N Engl J Med. 1999; 341: 427–434. 10.1056/NEJM199908053410607 10432328

[36] Thijs C, Knipschild P, Brombacher P. Serum lipids and gallstones: a case-control study. Gastroenterology. 1990; 99: 843–849. 2379787 10.1016/0016-5085(90)90978-a

[37] Halldestam I, Kullman E, Borch K. Incidence of and potential risk factors for gallstone disease in a general population sample. Br J Surg 2009; 96: 1315–1322. 10.1002/bjs.6687 19847878

[38] De Santis A, Attili AF, Ginanni Corradini S, Scafato E, Cantagalli A, De Luca C, et al Gallstones and diabetes: a case-control study in a free-living population sample. Hepatology. 1997; 25: 787–790. 10.1002/hep.510250401 9096577

[39] Seetahal SA, Bolorunduro OB, Sookdeo TC, Oyetunji TA, Greene WR, Frederick W, et al Negative appendectomy: a 10-year review of a nationally representative sample. Am J Surg. 2011; 201: 433–437. 10.1016/j.amjsurg.2010.10.009 21421095

[40] Chen DW, Davis RW, Balentine CJ, Scott AR, Gao Y, et al Utility of routine postoperative visit after appendectomy and cholecystectomy with evaluation of mobile technology access in an urban safety net population. J Surg Res. 2014; 190: 478–483. 10.1016/j.jss.2014.04.028 24880202

